# TIME SPENT FOR OTHERS: VARIED ASSOCIATIONS WITH WELL-BEING ACROSS TWO TIME SCALES AND SOCIODEMOGRAPHIC GROUPS

**DOI:** 10.1093/geroni/igae098.3755

**Published:** 2024-12-31

**Authors:** Kyoungmin Cho, David Almeida, Soomi Lee

**Affiliations:** PSU, state College, Pennsylvania, United States; Pennsylvania State University, University Park, Pennsylvania, United States; The Pennsylvania State University, University Park, Pennsylvania, United States

## Abstract

Existing research on spending time for others yields inconsistent findings regarding its effects on well-being. Some studies report positive effects, while others find negative effects. To address this inconsistency, our study explored whether spending time for others differentially impacts overall eudaimonic well-being and day-to-day hedonic well-being, considering potential differences across sociodemographic groups. 665 participants (Mage=48.27) in the Midlife in the United States Study completed both monthly survey and eight-day diary survey. Monthly and daily time spent for others, overall eudaimonic well-being (psychological and social domains), and daily hedonic well-being (positive affect, negative affect, daily stressors) were measured. OLS regression and multilevel models evaluated the associations of time spent for others with eudaimonic and hedonic well-being at the two time scales, adjusting for sociodemographic and health covariates (e.g., age, gender, education, self-reported physical health, marital status, day of week, and number of missed diary). Results indicated that, at the monthly level, participants who spent more time for others reported higher overall psychological and social well-being. However, at the daily level, they experienced more negative affect and stressors, with these effects more pronounced in men and adults under 56-year old. This study clarifies inconsistencies in prior research, highlighting differential associations of spending time for others with individual well-being across different time scales and sociodemographic groups. While short-term responses tend to be negative, particularly among men and younger individuals, long-term outcomes appear to be positive.

